# EFFECT OF ADVERSE CHILDHOOD EXPERIENCES ON DAILY SUPPORT TO FAMILY AND EMOTIONAL WELL-BEING IN ADULTHOOD

**DOI:** 10.1093/geroni/igz038.874

**Published:** 2019-11-08

**Authors:** Jooyoung Kong, Yin Liu, David Almeida

**Affiliations:** 1 University of Wisconsin-Madison, Madison, United States; 2 Utah State University, Logan, Utah, United States; 3 Penn State University, University Park, Pennsylvania, United States

## Abstract

Extensive evidence suggests that adverse childhood experiences (ACEs) can lead to negative health effects across a lifetime. This study examines the impact of ACEs on the frequency of providing daily support (i.e., unpaid assistance, emotional support, and disability-related assistance) to family members and the moderating effects of ACEs in the association between providing daily support to family and daily negative affect. Using the National Study of Daily Experiences II, we analyzed a total of 14,912 daily interviews from 2,022 respondents aged 56 on average. Key results showed that a greater number of ACEs were associated with providing more frequent emotional support to family. We also found the significant interaction effect that adults with more ACEs showed greater negative affect on the days when they provided assistance to family members with disabilities. The findings underscore the long-term negative impact of ACEs on daily well-being in the context of family relationships.

